# Chagas disease and its historicity

**DOI:** 10.1590/0074-02760210372chgsa

**Published:** 2022-05-16

**Authors:** Ana Carolina Vimieiro Gomes

**Affiliations:** 1 Universidade Federal de Minas Gerais

The article by Simone Kropf and Nísia Trindade Lima offers a path to reflections from the point of view of science history and social studies on the history of the discovery of Chagas disease (CD), whose 112th anniversary is being celebrated on the current special issue of Memórias do Instituto Oswaldo Cruz. Simone and Nísia are acknowledged researchers on the history of science and health in the first half of the twentieth-century in Brazil. Their major works “Doença de Chagas, doença do Brasil” (2009) and “Um sertão chamado Brasil” (1999), respectively, can be considered milestones and reference works for the renewal of the historiography of science and health in Brazil, after the 1990s. In the present article, the authors resume some of the basic interpretative pillars of their research agendas to think historically about how science is produced, how scientists act and how scientific knowledge is validated. To this end they mobilise five topics: the collective and social character of scientific activity; science as a practice and the various actions of scientists in their scientific work; the controversial character and the various paths to validate a scientific fact; and finally the importance of training and teaching new generations and forming networks of scientists to follow up on a given research agenda. By putting into perspective the science in action the history of the discovery of CD,^1^ ^)^ the authors propose to move away from the inventory of facts and anecdotes of the rituals of memory and commemoration in science.

The place of Simone Kropf and Nisia Lima’s article in a special topic entitled “Chagas disease: reflections of the past, challenges and opportunities for the future” is significant and determinant not just because it is the only one with a historical perspective. But, in addition to other articles currently dealing with CD regarding reflections on its status as a neglected disease and the difficulties in developing a vaccine, it’s epidemiological and clinical aspects, current forms of diagnosis and new drugs used in its treatment, the article by Kropf and Lima brings to the readers of the journal the dimension of the historicity of science around CD.

It is not only about history whatsoever, but about historicity. Historicity here is understood according to the definition of the historian of science of the Department of History of UFMG, Mauro Condé:^2^ “Science necessarily constitutes itself in a historical process not only in the chronological sense, that is, that takes place along the time, but also in the sense that the very history of a knowledge becomes a constitutive element of this knowledge and, thus, interferes in its final result. There is no knowledge without history and its history interferes in its results: what I call the historicity of science.” Following that reasoning, the current definition of CD would have its history contained in it. Epistemological, social, cultural and political aspects would integrate the changes by which the disease has gone through in its 112-year history ― as the authors have well demonstrated. The reflection on the historicity of CD is fundamental for the scientists themselves to understand what was known then and what is known about it today, what was done to validate it as a scientific fact and ― what are the future expectations to face the scientific challenges and public health problems still resulting from it. As Ludwik Fleck^3^ says “epistemology without historical and comparative investigations is no more than an empty play on words, or an epistemology of imagination (‘epistemologia imaginabilis’)”.

The five topics suggested by the authors are gateways to other potential themes and conceptual frames dear to the history of science. In the following part I will make general comments and highlight some possible issues to be considered on the agenda of reflections listed in the article.

Approaching “science as a collective enterprise” and “social activity” is one of the elementary grounds of analysis of the history and social studies of science. It is in the dynamic interaction between researchers among themselves and with the wider society that it is possible to realise how knowledge is a social act. In the article it is evident how CD is connected to the history of the Instituto Oswaldo Cruz (IOC) and its scientific, political, public health and national projects in the first decades of the 20th century. One can see how much Carlos Chagas being part of that group of researchers at the IOC was determinant for developing his scientific work. The IOC can be considered a place where they shared what science historian Lorraine Daston^4^ calls the moral economy of science, that is, a network of collective values and affections that would structure the ways scientists see knowledge. Moral economy of science helps us understand, for example, why researchers “dignify some objects of study at the expense of a great many others, trust some kinds of evidence and reject other sorts, and cultivate certain mental habits, methods of investigation, and even characters of a distinctive stamp”.

It is also worth mentioning the evidence brought by the authors for what I will call here interdisciplinarity, that is, the relevance of the transit and mutual influence of the network of researchers from various science fields and their different ways of knowing on Carlos Chagas’ studies on parasitic diseases: microbiology, tropical medicine, experimental medicine, clinical medicine, and even entomology with the knowledge about the blood-feeding insects. By the way, speaking of the transit of knowledge, I take this opportunity to highlight the central role of the circulation of science in knowledge changes and validation. I borrow the authors’ metaphor “winding road” to broaden their reflection to also encompass therein the issue about how local knowledge, that is, CD rooted in time and space, became “universal” and internationally recognized. Historians of science have been considering that knowledge becomes universal (consensually validated by peers) precisely because there is transit and a complex process of circulation within science and between science and society.

Finally, I highlight the powerful benefits of thinking of “science as a practice”. With this perspective we can perceive, for instance, how scientists act (scientifically and politically), the invisible actors and their invisible labor, the importance of scientific instruments and the new spaces for knowledge production. Here I emphasise the stimulating discussion brought by the authors about spatial turn in historiography and the “field science”* practiced by Carlos Chagas and in tropical medicine at the time. The “expedition to Lassange” has already become crystallised in the memory of the discovery of CD. We learned with the authors that the scientific expeditions to the “sertão” were part of the scientific culture and of the public health actions of the IOC. The field and their fieldwork merged with the spaces and practices of the IOC’s laboratories. It was difficult to distinguish “the field” and “the lab” in CD research. This displacement of space raises some questions, such as: How did Carlos Chagas and the researchers at the IOC and tropical medicine define the field and for what purposes? Which places, devices, infrastructures, and social relations were configured in their notion of field and fieldwork? How has the concept of field and fieldwork practices changed throughout the history of CD?

Simone Kropf and Nísia Trindade Lima’s article on the discovery of CD is therefore a strong invitation to scientists to engage with the History of Science issues and thus reflect on how science is experienced and humanly made, how it is transformed in time and space, and how the past has implications for the process of development of scientific knowledge today.

*On recent discussions on science and the concept of “field” see the Framing Statement of the workshop “What is a field”: https://web.sas.upenn.edu/whatisafield/what-is-a-field/.

Comments on the article: Kropf SP, Lima NT. The history of Chagas disease: reflections on science in action. Mem Inst Oswaldo Cruz. 2022; 117: e200372.

## References

[1] Latour B (2000). Ciência em ação: como seguir cientistas e engenheiros sociedade afora. Unesp.

[2] Condé MLL (2017). Um papel para a história: o problema da historicidade da ciência. Editora UFPR.

[3] Fleck L (1986). The problem of epistemology. In: Cognition and Fact.. Springer Nature.

[4] Daston L (1995). The moral economy of science. Osiris.

